# The Use of Triage in Primary Care in the UK: An Integrative Review and Narrative Synthesis

**DOI:** 10.1111/jan.70037

**Published:** 2025-07-01

**Authors:** Han Nah Park, Kyeongmin Lee, Carolyn Wallace

**Affiliations:** ^1^ University of South Wales Pontypridd Wales UK; ^2^ College of Nursing Seoul National University Seoul South Korea; ^3^ Wales Centre for Primary and Emergency Care Research Cardiff Wales UK

**Keywords:** integrative review, primary health care, triage

## Abstract

**Aims:**

To examine the use of triage systems in primary care in the UK.

**Design:**

Integrative literature review and narrative synthesis.

**Data Sources:**

PubMed, EMBASE, CINAHL and Cochrane Library were searched in October 2024.

**Methods:**

An integrative literature review was conducted following Whittemore and Knafl's (2005) five‐step process. Of 1440 articles retrieved, 305 duplicates were removed, and 1086 excluded after title and abstract screening. Two additional articles were identified through citation and hand searches. Twenty studies were quality‐assessed using the Mixed Methods Appraisal Tool, and data were extracted for narrative synthesis.

**Results:**

Twenty studies were selected, including four randomised controlled trials, three quasi‐experimental studies, eleven descriptive studies and two qualitative studies. The most common type of triage was telephone triage, most frequently performed by nurses. The most common health outcomes included subsequent patient re‐contacts after triage, patient symptoms or complaints, current health status and patient safety. The benefits of triage included high patient satisfaction, workload redistribution, reduced GP workload and emergency department crowding, improved resource utilisation, access to care and communication. The challenges of triage included increased overall contact time, mis‐triage issues, recruitment and retention challenges, the unsuitability of the decision support tool for the primary care setting and lower usage among older and less affluent groups.

**Conclusions:**

This review examined the current use of triage in primary care in the UK, identifying common patterns in triage processes and classifications. Several benefits were identified, though some ongoing concerns about triage remain.

**Impact:**

This paper provides essential evidence about the current use, effectiveness and challenges of triage in UK primary care. The findings can support healthcare policymakers, practitioners and researchers in planning and improving triage systems.

**No Patient or Public Contribution:**

Integrative review.

## 1 Introduction

Primary care is a comprehensive model that provides continuous, accessible and person‐centred care aiming to optimise population health and reduce health disparities. It integrates a wide range of services, including prevention, treatment and rehabilitation (WHO 2024). As the cornerstone of healthcare systems, primary care plays a vital and dynamic role in the United Kingdom (UK), serving as ‘the front door’ of the National Health Service (NHS). This includes general practice, community pharmacy, dental and optometry services (NHS England 2022a). The UK's primary care system, delivered through NHS, offers free healthcare services to all residents. All residents are required to register with a General Practitioner (GP), who acts as a gatekeeper by coordinating referrals to specialist and hospital care (NHS England 2024a). In recent years, demands for UK primary care is rapidly rising, driven by an aging population and the Coronavirus Disease 2019 (COVID‐19) pandemic (NHS England 2020). As this demand increases, addressing factors such as patient safety, satisfaction, managing practitioner stress, workload and health care costs have become central concerns (Eminovic et al. 2004; Hodgins et al. 2022; Gallagher et al. 1998; Kontopantelis et al. 2010; Orton et al. 2012; Richards et al. 2002). To manage these demands, triage systems have emerged as a crucial strategy in primary care.

Triage systems in primary care are structured processes for assessing the urgency and nature of patient needs and directing them to appropriate services (NHS England 2024b). These systems include care navigation by trained non‐clinical staff, clinical assessment by healthcare professionals and remote triage via telephone or digital platforms such as online consultations and messaging tools (Calitri et al. 2015; Eminovic et al. 2004; Holt et al. 2016; Horton et al. 2001). By enabling efficient allocation of care—through GPs, nurses, specialists or emergency services—triage helps prioritise patients based on clinical urgency and ensures timely and appropriate care (Campbell et al. 2014). By effectively directing patients to the right resources, triage systems help alleviate GP workloads, reduce healthcare costs, minimise waiting times and improve overall patient satisfaction and safety through more timely interventions (Campbell et al. 2014; Elliott et al. 2020; Gruffydd‐Jones et al. 2005; Richards et al. 2002). In practice, triage systems within UK primary care incorporate several classification types, such as advising self‐care, routine care or directing patients to community‐based or emergency services (Jiwa et al. 2002; Purc‐Stephenson and Thrasher 2012; Rolland et al. 2006). The specific classifications employed may vary depending on the clinical context and the organisational design of the service.

However, despite the potential benefits of triage in primary care, there remains a limited understanding of how these systems are implemented and used across UK settings, particularly in terms of their impacts on patient experience, healthcare costs, clinical efficiency, patient satisfaction and safety, as well as their associated challenges and benefits. Given the distinctive structure of the UK healthcare system and the diversity of triage models, a focused examination of UK‐based studies is essential to inform context‐specific policy and practice (NHS England 2024b). While existing studies have examined individual models, few provide an integrated understanding of their delivery, outcomes, benefits and challenges. This review addresses that gap by comprehensively examining the characteristics and implementation of triage systems in UK primary care.

### 1.1 Aims

This study aimed to examine the use of triage systems in primary care in the UK. It is designed to assess: (1) who conducts the triage, (2) the types of institutions that utilise triage, (3) what is the triage process, (4) what condition and which patients use triage, (5) whether a triage protocol, model or education/training exists, (6) the health outcomes used to evaluate the effectiveness of triage, (7) the benefits or challenges of triage in primary care and (8) the health economics of triage in primary care.

## 2 Methods

### 2.1 Design

An integrative literature review was adopted. We followed the five‐step process for conducting an integrative review outlined by Whittemore and Knafl's (2005), which includes problem identification, literature search, data evaluation, data analysis and presentation. Integrative reviews provide comprehensive and reliable results because they incorporate data from a variety of study designs. This approach is particularly valuable in the context of primary care, where the concept of triage is still relatively new. While systematic literature reviews can also include diverse study designs, we determined that an integrative review would better accommodate the methodological diversity in the literature and capture the evolving and multifaceted nature of primary care triage. Moreover, integrative reviews facilitate practical application in clinical settings by encompassing a broader range of relevant literature (Dhollande et al. 2021). The Preferred Reporting Items for Systematic Review and Meta‐Analyses guidelines and flow diagram were used for reporting to enhance transparency and ensure clear reporting of the review process (PRISMA, Page et al. 2021). The protocol was registered in PROSPERO (registration number: CRD42024601454).

### 2.2 Search Strategy

Search terms were established using the PICO‐SD (participants, intervention, comparison, outcome, study design) framework, with the assistance of a librarian. Main keywords were ‘primary health care’, ‘triage’ and ‘United Kingdom’. Search terms are shown in Appendix 1.

### 2.3 Information Sources

The electronic databases, including PubMed, EMBASE, CINAHL and Cochrane library, were searched on 14 October 2024. Database limits were English language. Citation and hand searching were carried out to retrieve additional studies.

### 2.4 Inclusion Criteria and Exclusion Criteria

The inclusion criteria were as follows: (1) Individuals who use or experience triage systems, or stakeholders such as funding organisations and commissions; (2) Studies that involve all methods of triage, including face‐to‐face triage, call triage and telehealth triage; (3) Studies conducted in a primary care or community setting in the UK; and (4) Studies written in English. No restrictions were applied regarding study design or time, but studies were limited to those conducted within the UK. Grey literature was not included in this review. The exclusion criteria were as follows: (1) Non‐peer‐reviewed articles; (2) Abstract‐only or inaccessible full texts.

### 2.5 Search Outcomes

**FIGURE 1:**
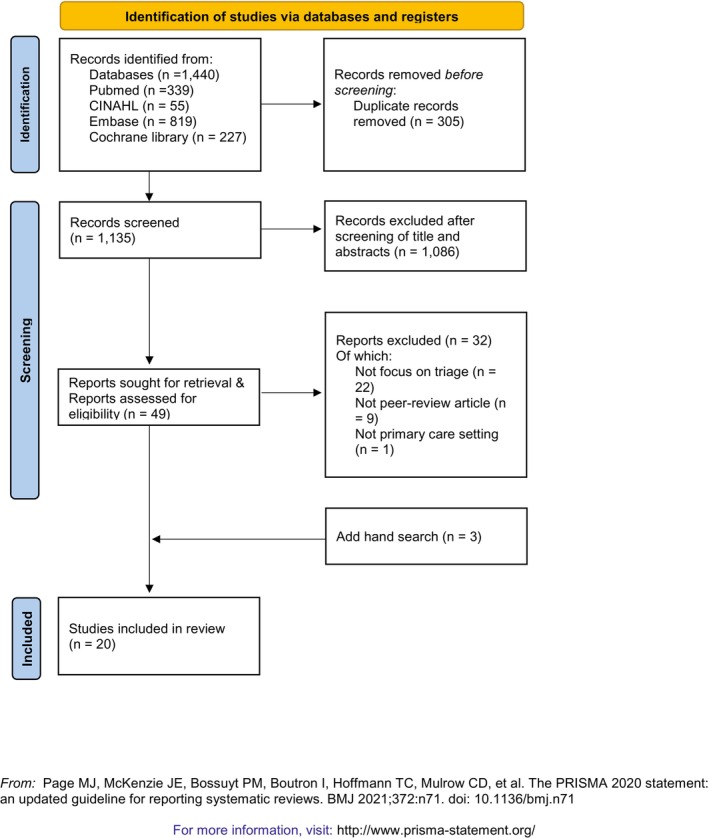
PRISMA flow chart for literature selection. From: Page et al. (2021). For more information, visit: http://www.prisma‐statement.org/.

Figure 1 shows the PRISMA flow diagram for this study (Page et al. 2021). Initially, 1440 articles were identified. After removing duplicates, 1135 studies remained. Through title and abstract screening, 1086 articles were excluded for not meeting the inclusion criteria. Subsequently, 49 articles underwent full‐text screening. Screening procedures were conducted independently by three reviewers (Park, Lee and Wallace), with disagreements resolved by Wallace. Of these, 32 articles did not meet the inclusion criteria. Three additional articles were included via hand searching. Thus, a total of 20 studies were included in this review.

### 2.6 Quality Appraisal

The Mixed Methods Appraisal Tool MMAT (Hong et al. 2018) was used to assess the quality of included studies. Compared to other appraisal tools such as CASP or JBI, we selected MMAT because it can evaluate multiple study designs within a single framework. This made it particularly suitable for our integrative review, which included diverse study designs. Quality appraisal was conducted independently by Park, Lee and Wallace, resolving discrepancies through discussion. The aim was to identify study strengths and weaknesses, not to exclude studies.

### 2.7 Data Extraction

The Covidence Programme was utilised for this integrative review. After searching four databases, results were imported into Covidence, which automatically removes duplicates (Covidence systematic review software 2025).

Park, Lee and Wallace screened titles and abstracts for eligibility, evaluating each as ‘yes,’ ‘no,’ or ‘maybe.’ When all reviewers agreed, the study proceeded to full‐text screening. During full‐text screening, we independently reviewed eligibility. Any disagreements were resolved by Wallace. All three reviewers had healthcare backgrounds. Wallace, a professor with extensive research experience, has over 40 years experience as nurse, nurse manager and academic/researcher. Park and Lee also have clinical nursing backgrounds and experience in healthcare research.

Key data from the included studies were extracted into a Microsoft Excel spreadsheet using a format designed by the research team. It included key study characteristics, such as the author, year, type of triage, type of triage provider, patient characteristics, triage process, triage protocol or model, benefits or challenges, health economics, triage outcomes, statistical methods and key findings.

### 2.8 Data Analysis and Synthesis

Due to differences in study designs and the varying quality of statistical data, a meta‐analysis was not feasible, and pooling results from different studies into a single, unified result was not possible. Therefore, a narrative synthesis was employed to explore relationships within and between studies. This approach was chosen as it facilitates the synthesis of diverse research questions, study designs and interventions (Popay et al. 2006).

The narrative synthesis followed four phases: theory development, developing a preliminary synthesis, exploring relationships within and between studies, and assessing the robustness of the synthesis (Popay et al. 2006). In the theory development phase, the necessity and relevance of triage in primary care were identified. In the preliminary synthesis phase, 20 studies were synthesised using an excel‐based data extraction form to capture core characteristics. Studies were grouped by common features such as triage type, provider, patient characteristics, process, protocol or model, benefits or challenges, health economics and outcomes. In the exploring relationships phase, researchers compared triage characteristics and outcomes across studies to identify patterns. Finally, in the assessing the robustness of the synthesis phase, the research team reviewed the finalised summary tables to evaluate the credibility and strength of the synthesis.

## 3 Result

### 3.1 Summary of the Included Studies

Twenty studies were identified for the final integrative literature review, and all selected studies were peer‐reviewed journal articles (Table 1). The studies were published between 1998 and 2022. The included studies were conducted in England (*n* = 16), Wales (*n* = 2), and Scotland (*n* = 2). The studies included 4 RCTs, 3 quasi‐experimental studies, 11 descriptive studies and 2 qualitative studies. Sample sizes ranged from 25 to 25,991,036. Three types of study settings were identified: 15 in GP practices or surgeries, 4 in call centres (NHS 24 [*n* = 2], NHS 111 [*n* = 2]) and 1 in a General Medical Practitioner (GMP) cooperative. The most common type of triage was telephone triage (*n* = 18), with the exception of one study using telephone and face‐to‐face triage together (Elliott et al. 2020) and another using web chat triage (Eminovic et al. 2004).

**TABLE 1:** Summary of included studies.

Author (year)	Study design	Aims	Setting	Participants	Triager	Data collection methods/Key measurements/Health outcome	Summary of findings related to triage
Calitri et al. (2015)	Descriptive study	To test whether satisfaction with same‐day care is positively associated with distance from practice for patients managed by GP or nurse telephone consultations	**England:** 42 GP practices in England (13 GP triage, 15 nurse triage, 14 usual care)	9154 patients, aged < 12 or ≥ 16: 1570 GP telephone; 1218 GP telephone + GP F2F; 316 GP telephone + Nurse F2F; 3429 GP F2F; 1649 Nurse telephone; 1649 Nurse telephone + GP F2F; 262 Nurse telephone + Nurse F2F	GP, Nurse	Satisfaction survey → Single questionnaire, 5‐point Likert scale	No overall link between satisfaction with same‐day care and distance. Both GP and nurse‐led telephone consultations were generally less satisfactory than F2F consultations. Patients managed by nurse consultation had the lowest satisfaction, which increased as the distance from the practice grew.
Campbell et al. (2014)	Cluster RCTs	To assess the effectiveness and cost implications of GP‐led and nurse‐led telephone triage compared to usual care for patients seeking same‐day consultations in primary care	**England:** 42 GP practices in England (13 GP triage, 15 nurse triage, 14 usual care)	16,211 patients, aged < 12 or ≥ 16: UC = 5572; GP‐led = 5171; Nurse‐led = 5468	GP, Nurse	Survey Primary: primary care contacts (general practice, out‐of‐hours primary care, A&E and walk‐in center attendances) in the 28 days following the index consultation request Secondary: resource use and costs, patient safety (deaths and emergency hospital admissions within 7 days of index request, A&E attendance within 28 days), health status and experience of care Health outcome: primary care contacts in the 28 days following the index consultation request, patient safety, health status of care	GP triage increased the mean number of contacts by 33%, and nurse triage by 48%, compared to usual care. Eight patients died within 7 days of the request: five in the GP‐triage group, two in the nurse‐triage group and one in the usual care group, with no relation to the trial groups. Economic evaluation: Despite more contacts, the estimated costs were similar across all groups (£75 per patient).
Charles‐Jones et al. (2003)	Qualitative study	To examine the perceptions of primary care staff regarding the purpose and impact of telephone triage by nurses on their clinical roles and professional identities	**England:** 9 practices in one health district in the North West of England	26 healthcare worker: 9GPs (6 M, 3 F, practice experience 3–28, mean 14.3), Practice nurses, Practice managers	Nurse	Semi‐constructive interview Discourse analysis	Four themes emerged from the data: (1) Justifying triage: the managerial benefits of controlling access and the potential benefits for the patient‐clinician relationship; (2) Categorising patients: patients are categorised based on their biomedical diagnoses and allocated to nurses or GPs; (3) Changing roles and identities: the allocation process strengthens the professional hierarchy within the practice; (4) Balancing conflicting aims: there is tension between the managerial need for biomedical triage and the aspiration for patient‐centred care.
Elliott et al. (2015)	Descriptive study	To examine the type, duration and outcome of the symptoms and health problems Scotland's nurse‐led telephone advice service (NHS 24) is contacted about and explore whether these vary by time of contact and patient characteristics	**Scotland:** NHS 24 (Call center) dataset, Scotland	NHS 24 data: 1,285,038 (1,061,347 out of hours call (82.6%), 223,691 in‐hour calls (17.4%))	Nurse	Secondary data analysis Proportion of cases by type, duration and outcome of symptoms and health problems for which NHS 24 is contacted. Health outcome: symptom and health problem type, duration, outcome	82.6% of calls were made out‐of‐hours, and 17.4% in‐hours. Abdominal issues were the most common reason for calls (12.2%), followed by dental (6.8%) and skin problems (6.0%). Call topics varied between in‐hours and out‐of‐hours. Most issues (62.9%) had started less than 24 h before contacting NHS 24, with shorter‐duration problems more common in out‐of‐hours calls. Out‐of‐hours callers were typically advised to visit an out‐of‐hours centre, while in‐hours callers were advised to contact their GP
Elliott et al. (2020)	Quasi‐experimental study	To examine if nurse‐led triage helps manage demand for GP appointments in primary care	**Wales:** 4 GP surgeries in the South Powys GP cluster in Wales (population 450,000)	Standard nurse‐led triage service = 24,060calls (2 year pilot) Total Nurse Triage service = 5298calls (6 months pilot) Patient satisfaction: 46 patients (female 30, male16)	Nurse	Clinical Outcomes (EMIS clinical system and manual records): The number of triage encounters undertaken; Type of triage encounter (phone, in person, via standard or total); triage outcome; age, sex Patient Satisfaction survey	In the standard nurse‐led triage service, 13,113 GP appointments were saved during the study period by offering advice, issuing prescriptions or ‘sick notes,’ or scheduling patients with a nurse. In the total nurse triage service, 2270 GP appointments were saved by redirecting patients to other services like dental, physiotherapy or community pharmacy. Overall, 93% of patients (*N* = 43) rated their experience as ‘excellent’.
Eminovic et al. (2004)	Descriptive study	To assess the opportunities and risks of a Web chat nurse triage service, focusing on its safety, feasibility and patient perceptions	**England:** Inner‐city GP practice in Coventry	25 patients (mean age 48 years; 57% female)	Nurse	Survey Time: web chat vs. NHS direct telephone consultations median duration Patient perception: Telemedicine Perception Questionnaire (TMPQ) instrument	In 45% of cases, nurses and GPs gave the same care recommendation. The CES nurse usually suggested a more urgent follow‐up, with only two cases where the nurse recommended a less urgent consultation. Web chat sessions had a median length of 30 min, twice as long as NHS Direct phone calls for similar cases. Patients' satisfaction with CES improved significantly, with many valuing the ability to revisit nurse responses and seeing CES as a helpful supplement to, but not a replacement for, regular care.
Gallagher et al. (1998)	Descriptive study	To determine the impact of telephone triage, conducted by a practice nurse, on the management of same day consultations in GP	**England:** GP in South Tyneside	▸11,300 patient; 1263 consultations recordings ▸postal survey 192	Nurse	Records and survey Changes in doctor and nurse workload, repeat consultations with the same problem, prescriptions issued, patient satisfaction with the service. Health outcome: repeat consultations with the same problem, prescriptions issued	Over 3 months, 1263 consultations were recorded, reducing doctor consultations by 54% (from 1522 to 664) compared to the prior 3 months. Nurses handled 26% (325) of doctor visit requests over the phone without an in‐person visit. In the surgery, 21% (273) saw only the nurse, 45% (565) saw only the doctor, and 8% (99) saw both. Between 29% and 41% patients made repeat consultations within 4 weeks. Of those surveyed, 88% were satisfied with the nurse's telephone advice. However, patients were unhappy about having to share their problem with the receptionist and waiting on the phone.
Gruffydd‐Jones et al. (2005)	Open RCTs	To examine the cost and effectiveness of targeted routine asthma care in GP using telephone triage, compared to usual clinic care	**England:** single semi‐rural practice in the southwest of England	146 athma patients (CG 62, IG 84)	Nurse	Survey Health outcome: Asthma Control Questionnaire (ACQ), health status and NHS resource utilisation	35% more patients (*n* = 84 vs. *n* = 62) received more than one consultation in the telephone group. Asthma control as measured by the ACQ was similar in the clinic and telephone groups. Economic evaluation: Mean NHS costs were £210 per patient per year in the telephone group compared to £334 in the clinic group
Hodgins et al. (2022)	Descriptive study	To evaluate the effectiveness of telephone triage in identifying urgent cases and hospital admissions for young adults with chest pain contacting NHS24	**Scotland:** Scotland‐wide Urgent Care Datamart (UCD), which links data from NHS24, the Scottish Ambulance Service, primary care out of hours (PCOOH), EDs, acute hospital admissions (Scottish Morbidity Records 01, SMR01), mental health admissions (SMR04) and National Records of Scotland death registrations	102,822 calls	Nurse	Second data analysis (retrospective study) Consultation pathways, Health outcome: acute inpatient admission, acute inpatient admission with an ‘acute‐ and‐ serious’ disease defined as discharge diagnosis of a condition requiring urgent treatment.	Out of 102,822 consultations, the most common pathway was an NHS24 call followed by a primary care out‐of‐hours (PCOOH) visit (37.6%). Hospital admissions occurred in 8.8% of cases, with 3.0% having a serious diagnosis. 8.2% received self‐care advice. Most cases ended at PCOOH (46.9%) or EDs (15.2%). Hospital admission rates were much higher for cases involving ambulance calls (28.7 times more likely) or a 1‐h GP home visit (36.8 times more likely), especially for serious diagnoses. ‘Asthma, unspecified’ was the most common serious diagnosis.
Holt et al. (2016)	Cluster RCTs	To determine whether telephone triage (GP‐led or nurse‐led) reduces clinician–patient contact time on the day of the request compared with usual care	**England:** 42 GP practices in England (13 GP triage, 15 nurse triage, 14 usual care)	1466 patient (692 UC, 302 GP triage, 472 nurse triage)	GP, Nurse	Records Overall composite clinician‐patient contact time on the index day (total time spent in contact with a GP or nurse: either through triage or F2F consultations) calculated based on mean duration and frequency	Telephone triage does not reduce overall clinician contact time (GP 4.0 min, nurse 6.6 min, usual care 9.5 min). Nurse‐led triage reduces GP contact time but leads to an overall increase in clinician contact time (GP 10.3 min, nurse 14.8 min, usual care 9.6 min)
Horton et al. (2001)	Quasi‐experimental study	To design and pilot a triage protocol that could be used by non‐dental staff to refer callers with dental complaints for appropriate treatment	**Wales:** GMP cooperative in North Wales, which provided an out‐of‐hours service for 61 GMPs	80 calls (3 months before triage 35, 3 months during triage 45)	Medical receptionist with training	Call records and structured telephone interviews (to evaluate patient opinions on the triage protocol) Call number, reason for call (Pt. symptom), action taken for callers with dental symptoms, opinions on the triage protocol Health outcome: Pt. symptom	Fewer dental callers used the out‐of‐hours service than expected. The triage protocol increased referrals to the GDP support line and provided more unregistered patients with dentist contacts. Although some had to call back, satisfaction improved after the protocol was introduced
Jiwa et al. (2002)	Descriptive study	To determine if telephone contact with a GP reduces the need for a F2F consultation where a patient initially suggests the need to see a GP on the same day	**England:** GP located in a market town, with a list size of 7200, and four GPs	3680 calls	GP	Survey Call duration and cost, demand for F2F appointment, Pt. satisfaction	A total of 3680 calls, with an average duration of less than two min (93% of calls lasting under 5 min). GP telephone triage reduced the demand for F2F appointments by 39%. While patients were satisfied with the service, the practice faced an additional cost of £200 per quarter in telephone charges
Jones et al. (1998)	Descriptive study	To survey the views of patients encountering nurse triage system for home visit requests, and to plot its effect on the routine visiting workload of doctors and availability at the sursery	**England:** GP in Gateshead, with a list size of approximately 10,000	1764 house calls 169 survey (1st 84, 2nd 85)	Nurse	Call records and Survey Triage outcome, Pt. satisfaction	1764 house call requests, with 41% doctor visit, 18% surgery consultation with doctor, 24% nurse advice given and accepted and 8% call passed to doctor for advice. 80% of respondents were satisfied with the help received from the nurse
Lewis et al. (2021)	Descriptive study	To evaluate patients compliance with NHS 111 advice	**England:** All NHS 111 caLLs in the Yorkshire and Humber region	3,631,069 calls were made to NHS 111 regarding adult patients	Nurse	Dataset: Connected Health Cities: Data linkage of urgent care data (CUREd) The proportion of callers in each disposition, percentage of callers attending ED within 4, 24 or 48 h of the NHS 111 caLL, time taken to attend ED, percentage of ED attendances classed as non‐urgent, percentage of ED attendances admitted	11% of patients (289,748) did not adhere to self‐care or visit primary care and went to the ED instead. Of these, 88% were classified as urgent and 37% were admitted to the hospital. Additionally, 9% of patients sent to the ED or ambulanced were deemed non‐urgent upon arrival. This highlights issues with mis‐triage and patient non‐compliance, affecting the accuracy and efficiency of triaging systems
Murdoch et al. (2015)	Qualitative study	To provide insights into the observed effects of the ESTEEM trial from the perspectives of staff, and to specify the circumstances under which triage is likely to be successfully implemented	**England:** 42 GP practices in England (13 GP triage, 15 nurse triage, 14 usual care)	Qualitative process evaluation: 4 GP triage, 4 Nurse triage Qualitative interview: 44 staff members in GP triage and nurse triage practices (16 GPs, 8 nurses, 7 practice managers, 13 administrative staff)	GP, Nurse	Qualitative semi‐structured interviews Staff perceptions and acceptability of triage, workload and resource allocation, communication and staff coordination	How triage impact: ○ Staff role: roles of doctors and nurses changed with triage ○ Workload: practices with enough staff felt triage worked better, while those with fewer staff felt overwhelmed ○ GP‐led triage: often resulted in uneven workload distribution—some doctors felt overwhelmed, while others had a lighter load ○ Nurse‐led triage: nurses using CDSS found it challenging to their clinical skills and reduced F2F patient care time, but some saw it as a learning opportunity that improved decision making
Nakubulwa et al. (2022)	Descriptive study	To investigate associations between compliance with advice given and patient and call characteristics	**England:** NHS 111 calls across London	1,964,726 patients of all ages for whom at least one call was made to the London NHS 111 helpline	Nurse	Call records Compliance with the advice given in the NHS 111 call	Callers followed advice in 49% of cases. Compliance was higher for calls about children under 16, women, people of Asian/Asian British ethnicity, and out‐of‐hours calls. The highest compliance was when self‐care was recommended without further healthcare contact
Richards et al. (2002)	Quasi‐experimental study	To compare the workloads of GPs and nurses and costs of patient care for nurse telephone triage and standard management of requests for same day appointments in routine primary care	**England:** Three primary care sites in York, England	4, 685 patients (1233 standard management, 3452 triage system)	Nurse	Computer records Type of consultation (telephone, appointment or visit), time taken for consultation, presenting complaints, use of services during the month after same day contact, and costs of drugs and same day, follow up and emergency care Health outcome: presenting complaints	The triage system reduced GP appointments by 29%–44%. It increased telephone care by 2.4 times, nurse care by 3.8 times and slightly reduced home visits by 15%. Triage took 1.7 min longer overall but saved 2.45 min of GP time. Nursing time increased, as did out‐of‐hours and A&E visits. Economic evaluation: Triage added an extra cost of £1.48 per patient, but this was not statistically significant.
Richards, et al. (2004a)	Descriptive study	To evaluate the accuracy and usefulness of audiotaping phone consultations to assess the decision quality of practice nurses triaging same‐day appointment requests	**England:** Three primary care sites in York, England	216 calls; Consultation: GP& nurse	Nurse	Call records and survey Information gathered (1 very poor‐5very good), appropriateness of the triage outcome (1 potentially dangerous, 2 safe but inappropriate, 3 safe and appropriate)	Triage nurses and assessors strongly agreed on identifying the problem, but agreement was moderate between GPs and nurse assessors on information gathered and outcome appropriateness. Information‐gathering was rated poor in 19.3% of calls and 3.2% were considered potentially dangerous. Reviewing 1% of consultations could identify unsafe calls 48% of the time and poor information‐gathering 99% of the time.
Richards, et al. (2004b)	Cluster RCTs	To compares the impact of off‐site NHS Direct triage and on‐site nurse triage on consultation workload and costs for same‐day appointment requests	**England:** Three primary care sites in York, England	4703 patients (2452 with practice based triage, 2251 with NHS Direct triage)	Nurse	Call records and survey Type of consultation after request for same day appointment (telephone, appointment or visit), time taken for consultation, service use during the month after same day contact, costs of same day, follow up and emergency care	Patients in the NHS Direct were less likely to have their call resolved by a nurse and more likely to see a GP. The average time per patient in the NHS Direct was 7.62 min longer. External triage by NHS Direct is feasible but more costly than practice‐based nurse triage. Economic evaluation: The average costs in the NHS Direct were £2.88 higher per patient compared to the practice‐based nurse triage due to differences in final point contact after triage.
Warren et al. (2015)	Descriptive study	To investigate associations between triage patients' demographic, health and lifestyle characteristics, and their reported experiences of care	**England:** 42 GP practices in England (13 GP triage, 15 nurse triage, 14 usual care)	12,132 patients (UC 4093; nurse triage 3704; GP triage 4,03)	GP, Nurse	Postal questionnaire survey Overall satisfaction with care (1 very satisfied‐5 very dissatisfied), ease of getting medical help or advice (1 very easy‐5 very difficult), convenience of care (1 very convenient‐4 not at all convenient)	Older patients reported higher overall satisfaction compared to those aged 25–59 years, while younger patients (aged 16–24) reported lower satisfaction. The response rate for overall satisfaction was lower in the nurse triage group (53%) compared to the usual care group (56%) and the GP triage group (60%). Patients who found it difficult to attend the practice reported lower satisfaction across all three trial groups

*Note:* A&E, Accident and Emergency; CG, control group; ED, emergency department; ESTEEM, embedded within a multicentre three armed cluster randomised controlled trial; F2F, face‐to‐face; GMP, general medical practitioner; GP, general practitioner; IG, intervention group; min, minutes; RCTs, Randomised Controlled Trial Study; UC, usual care.

The following subsections will provide a more detailed explanation of the key themes identified across these studies, including triage personnel, classification, patient symptoms, tools/protocols, education/training, health outcomes/health service use, benefits and challenges and health economics.

### 3.2 Quality Appraisal

Table 3 shows the study quality appraisal using MMAT version 2018. The studies included 4 RCTs (Campbell et al. 2014; Gruffydd‐Jones et al. 2005; Holt et al. 2016; Richards et al. 2004b), 3 quasi‐experimental studies (Elliott et al. 2020; Horton et al. 2001; Richards et al. 2002), 11 descriptive studies (Calitri et al. 2015; Elliott et al. 2015; Eminovic et al. 2004; Gallagher et al. 1998; Hodgins et al. 2022; Jiwa et al. 2002; Jones et al. 1998; Lewis et al. 2021; Nakubulwa et al. 2022; Richards et al. 2004a; Warren et al. 2015), and two qualitative studies (Charles‐Jones et al. 2003; Murdoch et al. 2015).

The four RCTs were of moderate to high quality. Gruffydd‐Jones et al. (2005) showed weaker adherence to baseline comparisons due to sex imbalance. All four studies were weaker in adherence to blinding, as outcome assessors were not blinded. Regarding intervention adherence, Campbell et al. (2014) and Gruffydd‐Jones et al. (2005) had low adherence rates (below 80%), and Holt et al. (2016) did not report this.

All three quasi‐experimental studies were of high quality, meeting most criteria (5 out of 7), with Richards et al. (2002) meeting all seven. However, Elliott et al. (2020) and Horton et al. (2001) were weaker in adherence to quality criteria for measurements and confounders, as they did not explain outcome measurements in detail or control for confounders.

The eleven descriptive studies were of moderate to high quality. Eight studies were high quality (*n* = 6: 6 out of 7; *n* = 2: 5 out of 7), while three were moderate (4 out of 7). Eminovic et al. (2004) and Gallagher et al. (1998) did not provide a sampling strategy, and Eminovic et al. (2004) and Jiwa et al. (2002) did not provide a representative population. Four studies did not clearly explain outcome measurements (Calitri et al. 2015; Gallagher et al. 1998; Jiwa et al. 2002; Richardset al. 2004a). All studies demonstrated weaknesses in adherence to nonresponse bias, With six studies not providing reasons for nonresponse (Calitri et al. 2015; Eminovic et al. 2004; Gallagher et al. 1998; Jiwa et al. 2002; Jones et al. 1998; Warren et al. 2015), and five studies relying on secondary datasets, making it impossible to determine nonresponse reasons (Elliott et al. 2015; Hodgins et al. 2022; Lewis et al. 2021; Nakubulwa et al. 2022; Richards et al. 2004a).

Two qualitative studies (Charles‐Jones et al. 2003; Murdoch et al. 2015) met all seven criteria.

### 3.3 Triage Personnel

In 13 of the 20 papers, triage was performed by nurses (Charles‐Jones et al. 2003; Elliott et al. 2015, 2020; Eminovic et al. 2004; Gallagher et al. 1998; Gruffydd‐Jones et al. 2005; Hodgins et al. 2022; Jones et al. 1998; Lewis et al. 2021; Nakubulwa et al. 2022; Richards et al. 2002; Richards et al. 2004a; Richards et al. 2004b); in 5 papers, by nurses or GPs (Calitri et al. 2015; Campbell et al. 2014; Holt et al. 2016; Murdoch et al. 2015; Warren et al. 2015); and in 1 paper each, by GPs (Jiwa et al. 2002) and medical receptionists (Horton et al. 2001).

Fourteen studies involved triage performed by individual nurses, doctors or medical receptionists, meaning these healthcare workers conducted the triage process alone without support from a team (Campbell et al. 2014; Charles‐Jones et al. 2003; Elliott et al. 2015; Eminovic et al. 2004; Gallagher et al. 1998; Gruffydd‐Jones et al. 2005; Hodgins et al. 2022; Holt et al. 2016; Horton et al. 2001; Jiwa et al. 2002; Jones et al. 1998; Lewis et al. 2021; Nakubulwa et al. 2022; Warren et al. 2015). In contrast, six studies involved team‐based triage, where healthcare workers worked together (Calitri et al. 2015; Elliott et al. 2020; Murdoch et al. 2015; Richards et al. 2002; Richards et al. 2004a; Richards et al. 2004b). The triage teams consisted of nurses and doctors (Calitri et al. 2015), nurses and receptionists (Murdoch et al. 2015; Richards et al. 2002; Richards et al. 2004a; Richards et al. 2004b) and nurses, doctors and receptionists (Elliott et al. 2020) (Table 1).

### 3.4 Triage Classification

Details of articles on triage classification are presented in Table 2. Among the 20 studies reviewed, 15 clearly presented a triage classification. Across these studies, triage levels were categorised as follows: self‐care advice or provision of health information; routine care or referral—including scheduled appointments or referrals to services such as A&E (Accident & Emergency, the UK emergency department [ED]), GPs, pharmacists, dentists, mental health practitioners and other NHS services; urgent care—requiring a same‐day appointment; emergency care—requiring an ED visit or a doctor's home visit; and life‐threatening—requiring an immediate response, such as a 999 caLL to dispatch an ambulance (the UK emergency number). While the terms urgent care and emergency care are used interchangeably, this review distinguishes between them due to the context of primary care triage. Urgent care refers to conditions requiring a same‐day GP appointment, whereas emergency care refers to more serious situations requiring immediate assessment at an ED or a doctor's home visit. Life‐threatening refers to critical conditions requiring immediate assistance from an ambulance.

**TABLE 2:** Characteristics of triage in primary care.

Author (year)	Triager/Method	Team or alone	Tool/Education or training	Patient characteristics and symptom	Triage pathway	Triage classification	Benefits or challenges
Calitri et al. (2015)	GP, Nurse/Telephone	Team (GP, nurse)	—	Most 25–59 years; Most female	Pt. calls practice seeking same‐day F2F consultation with GP → Computer Allocation (GP‐led triage, Nurse‐led, Usual care) → Triage	—	**Benefit:** Satisfaction with nurse telephone consultations rises by 7.2 points for every 10 km farther from the practice. **Challenge:** Patients prefer F2F over telephone consultations, and report lower satisfaction with nurse‐led care.
Campbell et al. (2014)	GP, Nurse/Telephone	Alone	**Tool:** Computer decision support in nurse triage: Odyssey patient assess **Education/Training:** Training workshops‐use of computer decision support software for practice nurses	Most female (60%), white (95%–97%), long‐standing health condition (46%–50%). ○ Mean age: UC 41.6 (SD 23.7), GP 44.7 (SD 25.0), Nurse 41.5 (SD 25.2).	Pt. calls practice seeking same‐day F2F consultation with GP → Computer Allocation (GP‐led triage, Nurse‐led, Usual care) → Triage (nurse using computer decision support tool)	(1) Self‐care advice (2) Further within‐practice consultation on the same day (3) Further within‐practice consultation on a subsequent day (4) Referral to another NHS service	**Benefit:** Redistribution of workload, cost neutrality, safety (the trial did not show evidence of increased deaths, hospital admissions or A&E visits due to triage) **Challenge:** Increased overall contact volume, reduced patient satisfaction, recruitment and retention issues
Charles‐Jones et al. (2003)	Nurse/Telephone	Alone	—	—	—	—	**Benefit:** Managerial benefits, improved resource allocation, workload redistribution **Challenge:** tension between efficiency and patient‐centred care
Elliott et al. (2015)	Nurse/Telephone	Alone	—	Most female (57.8%), under 35 years old, with usage declining above this age. commonly from affluent, urban areas, especially in central belt regions. Top Problems: → Out‐of‐Hours: Abdominal, Rash/Skin, Breathing → In‐Hours: Dental, Abdominal, Medication—Total: Abdominal, Dental, Rash/Skin. Common Problems by Age Group: → Infants (0–1): Baby/infant issues, Rash/Skin—Children (1–4): Rash/Skin, Abdominal → Teens and Young Adults (16–24): Abdominal, Chest Pain → Adults (25–64): Abdominal, Dental → Older Adults (65): Abdominal, Breathing, Genito‐urinary	Pt. call → NHS nurse triage	(1) Self‐care advice or provide health information (2) Clinical follow‐up: doctor/nurse/service clinician will call Pt. for further assessment or advice (3) Referral (to A&E, GPs, pharmacists, dentists, mental health practitioners) (4) Home visit by doctor (5) call 999	**Benefit:** Timely intervention **Challenge:** Lower usage of NHS 24 by older and less affluent groups
Elliott et al. (2020)	Nurse/Telephone or F2F triage	Team (nurse, GP, receptionist)	**Tool:** Egton Medical Information Systems (EMIS) Web clinical system—template	Standard: 14,017 female (58%), 10,043 male (42%) Total: not mentioned	Standard nurse‐led triage: Pt. visit or call to request same‐day appointments → RN assess and triage Pt. Total nurse triage: Patient call to request same‐day or routine appointments → RN assess and triage Pt.	(1) Advice only (2) Appointment with nurse (3) Routine GP appointment (4) Same‐day appointment with GP (5) Prescription or ‘sik note’ (6) Referral for further care (7) GP home visit (8) Emergency 999 call	**Benefit:** reduced GP workload, efficient resource utilisation, high satisfaction level of patient **Challenge:** Nurse confidence and competence, resource and staffing limitations
Eminovic et al. (2004)	Nurse/Web chat	Alone	**Tool:** Web chat, NHS Clinical Assessment System (NHS CAS), a triage decision support system generating questions and advice based on patient answers, used in the telephone NHS Direct service **Education/Training:** 5 NHS Direct‐trained nurses based in Southampton were further trained in the use of Web chat	Non‐urgent patients (ie, those telephoning the practice who did not request an appointment that day) Symptoms: elbow pain; back pain; breast lump; chest pain; couth; ear problems; fatigue; knee pain/swelling; neck injury; rach; sore throat; urine frequency	Pt. in practice waiting room → Web chat triage	(1) Home care (2) Contact your GP within 4, 12 or 36 h or within 2 weeks (3) Visit the A&E department within 4 h (4) Visit the A&E department as soon as possible (5) Call 999 for an urgent ambulance	**Benefit:** Consistent care recommendations, improved patient satisfaction **Challenge:** Longer session duration, not a replacement for regular care
Gallagher et al. (1998)	Nurse/Telephone	Alone	**Tool:** Developed written guidelines, in association with all doctors and nurses, for the management of acute conditions such as cystitis, sore throat, and vaginal discharge **Education/Training:** Training in consultation techniques (learned by sitting in with the doctors) and in diagnosing and treating acute and chronic illness	—	Pt. call requesting to see a doctor → Nurse triage	(1) Telephone advice only (2) Consultation with the nurse (3) Consultation with the doctor (4) Consultation with the nurse and doctor (5) consultation with the physiotherapist	**Benefit:** Reduced doctor consultations, 88% patients were satisfied with the nurses' telephone advice **Challenge:** Repeat consultations, receptionist involvement, telephone waiting time
Gruffydd‐Jones et al. (2005)	Nurse/Telephone	Alone	—	Adult patients with asthma CG: mean age 49.6 (SD 16.1), male 38, female 59, asthma mild/moderate 92, severe 5 IG: mean age 50.8 (SD 15.4), male 50, female 47, asthma mild/moderate 91, severe 6	Nurse call Pt. at 6‐monthly intervals→categorise Pt. as high or low risk→formulate individualised asthma action plan: ▸Low risk: individualised asthma action plan with advice ▸High risk: clinic asthma review arrange→if asthma control deemed stable for 3 months, patients were returned to telephone review	Low risk: individualised asthma action plan with advice High risk: clinic asthma review arrange	**Benefit:** Cost savings, improved patient access, high levels of patient satisfaction
Hodgins et al. (2022)	Nurse/Telephone	Alone	—	All callers aged 15–34 years to NHS24 with ‘chest pain’ recorded as the call reason (chest pain including traumatic and non‐traumatic causes) 36.9% male, 63.1% female	Pt. call→nurse triage	(1) Self‐care advice given (2) Schedule daytime appointment (3) Primary care out of hours visits (4) Attend emergency department (5) Call 999 (ambulance)	**Benefit:** Effective triage assessment, reduced burden on emergency services, safety and reliability **Challenge:** Uncertainty of diagnosis
Holt et al. (2016)	GP, Nurse/Telephone	Alone	**Tool:** Computer decision support software	UC: 274 male, 418 female GP triage 120 male, 182 female nurse traige: 184 male, 288 female	Pt. calls practice seeking same‐day F2F consultation with GP → Computer Allocation (GP‐led triage, Nurse‐led, Usual care) → Triage (nurse using computer decision support tool)	—	**Benefit:** Reduced GP time in nurse‐led triage **Challenge:** No overall time savings, extended F2F consultations
Horton et al. (2001)	Medical receptionist with training/Telephone	Alone	**Tool:** Figure 1. The triage protocol (94p)	Dental pain 47 (59%), dental pain and swelling 16 (20%), swelling 13 (16%), bleeding 2 (3%), no symptoms recorded 2 (3%)	Pt. call evening and weekend on‐call service in GMP cooperative at Deeside → Medical receptionist triage using triage protocol	(1) Basic self‐care advice (2) Referral to a dentist as soon as possible within normal working hours (their own dentist for those patients who were registered and a named dentist accepting NHS patients, provided by the North Wales Health Authority, for those who were unregistered) (3) Referral for immediate emergency treatment at the nearest district general hospital (4) Referral for advice to a GDP support line (This option was to be used where the medical receptionist had difficulty in interpreting the patient's history or the severity of their dental problem)	**Benefit:** Improved Pt. satisfaction, efficient resource use, support for unregistered patients **Challenge:** 23% referred GDP support line due to uncertainty about Pt. problems
Jiwa et al. (2002)	GP/Telephone	Alone	—	—	Pt. request same‐day appointments → GP call pt. and triage	(1) Advice only (2) Routine appointment offered (not same day) (3) Appointment with nurse (4) Prescription offered without F2F consultation (5) Same day appointment offered (6) Visit	**Benefit:** Reduced demand for F2F appointment, high patient satisfaction (98%), increased efficiency **Challenge:** Increased telephone costs (26% more)
Jones et al. (1998)	Nurse/Telephone	Alone	**Tool:** The computerised record of the patient concerned was displayed during the telephone triage process to facilitate the consultation	—	Pt. call request house visit → Practice nurse triage using computer	(1) Nurse advice given and accepted (2) Call passed to doctor for advice (3) Surgery consultation with doctor (4) Doctor to visit	**Benefit:** High patient satisfaction (82%) **Challenge:** Concerns about the nurse triage (prefer doctor visit, whether nurses were qualified to decide when a doctor's visit was necessary)
Lewis et al. (2021)	Nurse/Telephone	Alone	—	Pt. aged 16 or over	Pt. call NHS 111 → Nurse triage	(1) Self care (2) refer other service: local service, mental health services, district nurse, midwife (3) refer primary or community care: GP, dental practitioner, community pharmacy, within a given time frame (4) Attend ED (5) Ambulance dispatch: call 999	**Benefit:** 68.6% Pt. advised to attend the ED complied, reduced ED overcrowding (76.2% calls advised for self‐care or primary care rather than ED) **Challenge:** Pt. non‐compliance (Pt. advised to self‐care or primary care, 11% Pt. went to ED, among 88% were classified as urgent, 37% admitted), mis‐triage and over‐cautious recommendation
Murdoch et al. (2015)	GP, Nurse/Telephone	Team (receptionist, nurse)	**Tool:** Computer decision support in nurse triage: Odyssey patient assess	—	Pt. call GP →Receptionist: if Pt. requesting same‐day or F2F appointment with a GP, ask phone number →GP or nurse call back 1‐2 h later, triage using computerised decision support tool	—	**Benefit:** Reduced GP workload, improved efficiency in patient care, nurse developed clinical decision‐making skills, improved Pt. access, enhanced communication (regular meetings, feedback) **Challenge:** Increased workload (nurse particular), nurse felt their clinical skills were underused because F2F care replaced by telephone‐based assessment, CDSS unsuitable for primary care
Nakubulwa et al. (2022)	Nurse/Telephone	Alone	—	Women (58%), adults aged 30–59 years (33%) and people in the white ethnic category (36%)	Pt. call NHS 111 → Nurse triage	(1) Self‐care (2) Advised to Attend Other Service (3) Advised to Attend Primary and Community Care (4) Advised to Attend ED (5) Ambulance Dispatches	**Benefit:** Increased access to care, efficient use of resources **Challenge:** low compliance (only 49% of callers complied with the triage advice), inconsistent compliance across advice types (compliance: 67% ambulance, 43% ED, 35% primary and community care)
Richards et al. (2002)	Nurse/Telephone	Team (receptionist, nurse)	**Tool:** Computerised management protocol developed by the practice **Education/Training:** 30 h of minor illness management training	Standard management: male 480 (38.9), female 753 (61.1) Triage system: male 1361 (39.4), female 2091 (60.6)	Pt. request a same day appointment → Receptionist → Standard management or triage using computerised management protocol	(1) Telephone advice only (2) Routine nurse or GP appointment (3) Same day nurse appointment (4) Same day GP appointment (5) Home visit	**Benefit:** Reduced GP workload, efficient use of nurse‐led care **Challenge:** Increased overall time and nursing workload, higher rates of follow‐up, out‐of‐hours, ED visits
Richards et al. (2004a)	Nurse/Telephone	Team (receptionist, nurse)	**Tool:** Computerised management protocol developed by the practice **Education/Training:** 30 h of in‐house education on minor illness management	60.1% women, with an average age of 37.2 years. Slightly higher standardised mortality ratio, unemployment and proportion of pensionable residents than the regional UK average	Pt. request a same day appointment →Receptionist →Practice nurse triage using computerised management protocol	(1) Immediate telephone advice only to patient and/or carer (2) Telephone call with a GP (3) Routine appointment with a practice nurse on another day (4) Routine appointment with a GP on another day (5) Appointment with the triage nurse on the same day (6) Appointment with a GP on the same day (7) Home visit from a GP	**Benefit:** Nurse managed 38.1% calls without GP involve **Challenge:** Inconsistent decision quality, risk of unsafe decisions (3.2% calls were considered potentially unsafe)
Richards et al. (2004b)	Nurse/Telephone	Team (receptionist, nurse)	**Tool:** NHS Direct computerised decision‐making algorithms	NHS Direct (*n* = 2260): male 859 (38.0), female 1401 (62.0), Mean number of complaints 1.26 (0.56) Practice (*n* = 2458): male 899 (36.6) female 1559 (63.4), Mean number of complaints 1.23 (0.54)	Pt. request a same day appointment → Receptionist → Triage using NHS direct computerised decision‐making algorithms by NHS triage nurse or practice triage nurse	(1) Telephone advice only (2) Routine nurse appointment (3) Routine GP appointment (4) Same day nurse appointment (5) Same day GP appointment (6) Home visit	**NHS direct triage Challenge:** NHS triage took 7.62 min longer and cost £2.88 more per patient than practice triage. NHS triage also referred more patients to GPs, while practice triage resolved more cases with nurses.
Warren et al. (2015)	GP, Nurse/Telephone	Alone	—	More female (> 60%); mostly 25–59 years (> 39%); Deprivation (5 ~ 6.4% lowest for quantile 5‐ most deprived); mostly no longterm conditions (49.9% ~ 54%)	—	—	**Benefit:** High Pt. satisfaction (nurse triage, GP triage, usual care), ethnic minorities reported high satisfaction with GP triage, most deprived group reported greater convenience in accessing care **Challenge:** Satisfaction lower for younger aduts (16–24)

Abbreviations: CG, control group; F2F, face‐to‐face; GMP, General Medical Practitioner; GP, general practitioner; IG, intervention group; Pt., patient; RN, registered nurse.

**TABLE 3:** Quality assessment using MMAT.

Author (year)	Category of study designs	Item						
		S1	S2	1	2	3	4	5
Calitri et al. (2015)	Quantitative study; descriptive study	Y	Y	Y	Y	N	N	Y
Campbell et al. (2014)	Quantitative study; Cluster RCTs	Y	Y	Y	Y	Y	N	N
Charles‐Jones et al. (2003)	Qualitative study	Y	Y	Y	Y	Y	Y	Y
Elliott et al. (2015)	Quantitative study; descriptive study	Y	Y	Y	Y	Y	C	Y
Elliott et al. (2020)	Quantitative study; Quasi‐experimental design	Y	Y	Y	N	Y	N	Y
Eminovic et al. (2004)	Quantitative study; descriptive study	Y	Y	N	N	Y	N	Y
Gallagher et al. (1998)	Quantitative study; descriptive study	Y	Y	C	Y	N	N	Y
Gruffydd‐Jones et al. (2005)	Quantitative study; Open RCTs	Y	Y	Y	N	Y	N	N
Hodgins et al. (2022)	Quantitative study; descriptive study	Y	Y	Y	Y	Y	C	Y
Holt et al. (2016)	Quantitative study; Cluster RCTs	Y	Y	Y	Y	Y	N	C
Horton et al. (2001)	Quantitative study; Quasi‐experimental design	Y	Y	Y	N	Y	N	Y
Jiwa et al. (2002)	Quantitative study; descriptive study	Y	Y	Y	N	N	N	Y
Jones et al. (1998)	Quantitative study; descriptive study	Y	Y	Y	Y	Y	N	Y
Lewis et al. (2021)	Quantitative study; descriptive study	Y	Y	Y	Y	Y	C	Y
Murdoch et al. (2015)	Qualitative study	Y	Y	Y	Y	Y	Y	Y
Nakubulwa et al. (2022)	Quantitative study; descriptive study	Y	Y	Y	Y	Y	C	Y
Richards et al. (2002)	Quantitative study; Quasi‐experimental design	Y	Y	Y	Y	Y	Y	Y
Richards et al. (2004a)	Quantitative study; descriptive study	Y	Y	Y	Y	N	C	Y
Richards et al. (2004b)	Quantitative study; Cluster RCTs	Y	Y	Y	Y	Y	N	Y
Warren et al. (2015)	Quantitative study; descriptive study	Y	Y	Y	Y	Y	N	Y

*Note:* Item S1: Clear research questions; Item S2: Appropriate data for research question; Quantitative randomised controlled trials methodological quality criteria includes Item 1: Randomizations, Item 2: Baseline comparisons, Item 3: Complete outcome, Item 4: Blinding, Item 5: Intervention adherence; Quantitative non‐randomised methodological quality criteria includes Item 1: Representative population, Item 2: Measurements, Item 3: Complete outcome, Item 4: Confounders, Item 5: Intervention administered as intended; Quantitative descriptive methodological quality criteria includes Item 1: Sampling strategy, Item 2: Representative population, Item 3: Measurements, Item 4: Nonresponse bias, Item 5: Statistical analysis; Qualitative methodological quality criteria includes Item 1: Approach Suitability, Item 2: Data Collection Adequacy, Item 3: Data‐Derived Findings, Item 4: Data‐Substantiated Interpretation, Item 5: Coherence and Consistency.

Abbreviations: C, cannot tell; MMAT, mixed‐method assessment tool; N, No; RCTs, randomised controlled trial study; Y, yes.

Based on this framework, four types of triage classification were identified across the studies. The three‐class system (*n* = 3: Campbell et al. 2014; Gallagher et al. 1998; Gruffydd‐Jones et al. 2005) included self‐care advice, routine care or referral and urgent care. The four‐class system without a 999 call (*n* = 6: Horton et al. 2001; Jiwa et al. 2002; Jones et al. 1998; Richards et al. 2002; Richardset al. 2004a; Richardset al. 2004b) comprised self‐care advice, routine care or referral, urgent care and emergency care. The four‐class system with a 999 call (*n* = 5: Elliott et al. 2015; Eminovic et al. 2004; Hodgins et al. 2022; Lewis et al. 2021; Nakubulwa et al. 2022) included self‐care, routine care or referral, emergency care and life‐threatening. Finally, the five‐class system (*n* = 1: Elliott et al. 2020) covered self‐care advice, routine care or referral, urgent care, emergency care and life‐threatening.

### 3.5 Symptoms in Patients Using Triage in Primary Care

The most common symptoms reported in triage calls in primary care were abdominal, dental and skin problems, with variations based on call time and age group (Elliott et al. 2015). Triage for mild to moderate asthma was commonly performed in primary care (Gruffydd‐Jones et al. 2005). Additionally, patients using evening and weekend dental on‐call services presented with dental pain, dental pain with swelling, swelling alone and, less commonly, bleeding or unspecified symptoms (Horton et al. 2001).

### 3.6 Tool or Protocol

There are 11 out of 20 studies using a tool or protocol such as a computer decision support tool (Campbell et al. 2014; Eminovic et al. 2004; Holt et al. 2016; Murdoch et al. 2015), computerised management protocol (Richards et al. 2002; Richards et al. 2004a; Richards et al. 2004b), web clinical system template (Elliott et al. 2020; Jones et al. 1998), written guidelines (Gallagher et al. 1998) and triage protocol (Horton et al. 2001).

### 3.7 Education or Training

Out of the 20 studies reviewed, five included descriptions of triage‐related education or training programmes (Campbell et al. 2014; Eminovic et al. 2004; Gallagher et al. 1998; Richards et al. 2002; Richards et al. 2004a). Campbell et al. (2014) described training workshops focused on using computer decision support software specifically designed for practice nurses. Eminovic et al. (2004) NHS Direct‐trained nurses received additional training in web chat triage. Gallagher et al. (1998) described training in consultation techniques, which included shadowing doctors to gain hands‐on experience in diagnosing and managing both acute and chronic illnesses. In Richards et al. (2002) and Richards et al. (2004a), the training consisted of a 30‐h programme in minor illness management.

### 3.8 Health Outcomes and Health Service Use in Evaluating the Effectiveness of Triage in Primary Care

Seven out of the 20 studies used health outcomes and health service use as a measure of triage effectiveness. Health outcomes included patient symptoms or complaints (Elliott et al. 2015; Horton et al. 2001; Richards et al. 2002) and current health status (Gruffydd‐Jones et al. 2005). Health service use included patient re‐contacts after triage (Campbell et al. 2014; Elliott et al. 2015; Gallagher et al. 1998; Hodgins et al. 2022), such as primary care contacts within 28 days following the index consultation request (Campbell et al. 2014), repeat consultations for the same problem (Gallagher et al. 1998), and acute inpatient admissions (Hodgins et al. 2022).

### 3.9 Benefits and Challenges of Triage in Primary Care

Table 2 presents the benefits and challenges of primary care triage as identified in the selected studies. The benefits include high patient satisfaction (Calitri et al. 2015; Elliott et al. 2020; Eminovic et al. 2004; Gallagher et al. 1998; Gruffydd‐Jones et al. 2005; Horton et al. 2001; Jiwa et al. 2002; Jones et al. 1998; Warren et al. 2015), workload redistribution (Campbell et al. 2014; Charles‐Jones et al. 2003; Hodgins et al. 2022) and reduced GP workload (Elliott et al. 2020; Gallagher et al. 1998; Holt et al. 2016; Jiwa et al. 2002; Murdoch et al. 2015; Richards et al. 2002; Richards et al. 2004a). It also reduces the burden on emergency services (Hodgins et al. 2022), alleviates ED crowding (Lewis et al. 2021) and enables more efficient resource utilisation (Charles‐Jones et al. 2003; Elliott et al. 2020; Horton et al. 2001; Nakubulwa et al. 2022). Triage also supports patient safety (Eminovic et al. 2004; Hodgins et al. 2022) through consistent care recommendations (Eminovic et al. 2004), improves patient access to care (Gruffydd‐Jones et al. 2005; Horton et al. 2001; Murdoch et al. 2015; Nakubulwa et al. 2022), offers cost savings (Gruffydd‐Jones et al. 2005) and enhances communication through regular meetings and feedback (Murdoch et al. 2015). Additionally, triage allows for timely interventions (Elliott et al. 2015).

The challenges include increased overall contact time (Campbell et al. 2014; Eminovic et al. 2004; Holt et al. 2016; Murdoch et al. 2015; Richards et al. 2002) and higher nurse workload (Murdoch et al. 2015; Richards et al. 2002). Mis‐triage (Gallagher et al. 1998; Hodgins et al. 2022; Horton et al. 2001; Lewis et al. 2021; Richards et al. 2004a) was another concern, including inconsistent decision‐making quality (Richards et al. 2004a) and repeat consultations (Gallagher et al. 1998). There were also concerns about nurse triage, with some patients preferring to consult a doctor (Jones et al. 1998; Calitri et al. 2015). Some nurses reported low confidence and competence in triage (Elliott et al. 2020), while other nurses felt their clinical skills were underused because telephone‐based assessment replaces face‐to‐face care (Murdoch et al. 2015). Other challenges included recruitment and retention issues (Campbell et al. 2014; Elliott et al. 2020), low patient compliance (Lewis et al. 2021; Nakubulwa et al. 2022) and the unsuitability of computer decision support systems (CDSS) for primary care (Murdoch et al. 2015). Patients also preferred face‐to‐face consultations over telephone triage (Calitri et al. 2015), and long telephone waiting times (Gallagher et al. 1998) were also a concern. Additionally, the utilisation of Scotland's telephone advice service (NHS 24) among older and less affluent groups was lower (Elliott et al. 2015).

### 3.10 Health Economic Evaluation of Triage in Primary Care

Four of the 20 studies assessed health economic evaluations to assess triage effectiveness (Campbell et al. 2014; Gruffydd‐Jones et al. 2005; Richards et al. 2002; Richards et al. 2004b). Campbell et al. (2014) compared GP‐led and nurse‐led telephone triage with usual care for same‐day consultations in primary care, finding similar costs of around £75 per patient across all groups despite more contacts in the triage groups. Gruffydd‐Jones et al. (2005) reported lower annual NHS costs for telephone‐based asthma care (£210 per patient) compared to clinic‐based care (£334). Richards et al. (2002) examined the costs of nurse‐led telephone triage compared to standard management for same‐day appointment requests in primary care. Triage added a marginal extra cost of £1.48 per patient, though this difference was not statistically significant. Finally, Richards et al. (2004b) compared off‐site NHS Direct triage with on‐site nurse triage for same‐day appointment requests. NHS Direct triage incurred an additional cost of £2.88 per patient, largely due to differences in follow‐up contact points after triage.

## 4 Discussion

In this integrative review, we examined the current use of triage in the UK primary care, identifying key trends, challenges and gaps. The review found triage is predominantly nurse‐led, resulted in work redistribution and involves variable triage classifications. While triage improves access and resource use, challenges remain‐such as mis‐triage, inconsistent training and education, limitations in guidelines and decision support tools. These findings have implications for how triage is implemented, evaluated and supported in the future.

We found that most triage in primary care was conducted by nurses. This aligns with a previous systematic literature review examining the effects of telephone consultation and triage. One reason for introducing triage in primary care is to reduce GP workload (Bunn et al. 2005), which may explain the rise in nurse‐led triage. Nurse‐led triage reallocates some workload from GPs to nurses, thus reducing GP workload and potentially improving GP availability (Campbell et al. 2014; Holt et al. 2016; Jiwa et al. 2002; Lewis et al. 2021; Nakubulwa et al. 2022). On the other hand, GP‐led triage could potentially increase GPs' workload instead of alleviating it. Simply shifting clinicians into triage roles is unlikely to release them from other tasks (Mayor 2014).

However, there still remains a question as to whether workload redistribution is better. Both GP‐and nurse‐led telephone triage did not reduce total clinician contact time compared to usual care (Holt et al. 2016) and increased the number of contacts after triage within 28 days, which ultimately increased overall workload (Campbell et al. 2014). While nurse‐led triage reduced GP time, the overall clinician (GPs and nurses) time increased (Holt et al. 2016) as triage redistributed workload from GPs to nurses and from face‐to‐face to telephone rather than reducing it. For nurses, this work redistribution leads to a new role of triage, which has raised concerns about their ability to provide appropriate triage on patients, leading to low confidence (Elliott et al. 2020). Some nurses expressed concerns that transitioning from face‐to‐face to telephone triage prevented them from effectively demonstrating their clinical skills (Murdoch et al. 2015). Successful workload redistribution requires clear guidelines for newly allocated work, staff education and training and the provision of decision support tools to assist triage decision‐making.

Triage classifications varied across studies, but all studies included self‐care or information providing, routine care or referral and urgent/emergency care. The lack of standardisation across primary care settings complicates the development of clear guidelines or protocols. While there is limited research specifically on primary care triage classifications, studies on ED triage provide useful insights. According to international literature, despite variations in implementation, EDs rely on validated triage scales tailored to their specific contexts (Farrohknia et al. 2011). Meanwhile, although there is no validated and standardised triage scale in primary care yet, research is ongoing. In the Netherlands, the Netherlands Triage Standard (NTS) is employed in both ED and after‐hours primary care, with ongoing research into its effectiveness (Erkelens et al. 2020). Australia and New Zealand have implemented national telephone triage services using standardised decision‐support software (St George et al. 2008), and England, Scotland and Wales introduced NHS 111 (NHS England 2024c). These international examples reflect increasing global efforts to standardise triage classifications in primary care. In this context, our study contributes valuable groundwork for informing future guideline and protocol development.

The successful implementation of triage depends on the clinical skills of the practitioner and the decision support tools available (Fekonja et al. 2023). In primary care, where staff shortages and recruitment challenges are common (Lawson 2022), finding experienced and skilled staff can be difficult. Decision support tools for triage provide knowledge, patient‐specific information and recommendations, helping ensure effective and safe decision‐making (Michel et al. 2024). Nurses using these tools reported feeling more competent and confident in their assessments, as the tools helped confirm or refine their knowledge. However, current clinical decision support systems are not designed for primary care, making it challenging to address specific cases, such as skin conditions (Murdoch et al. 2015). Using decision support tools or protocols for triage not only reduces the psychological burden on medical staff but also enhances the effectiveness and safety of triage. This emphasises the need to develop triage tools or protocols for primary care settings.

Education or training is another key factor for triage. Education or training ensure consistent and accurate triage (Butler et al. 2023) and help prepare staff effectively for this role (Crouch et al. 1997). Education is simple, cost‐effective and easy to implement, with a proven positive impact on both the behaviour and knowledge of the staff involved in triage (Butler et al. 2023). However, in this review, only 5 out of 20 studies described education or training, and even in those, the content was not presented in sufficient detail to allow meaningful comparisons. In the absence of widely accepted standards for primary care triage, this review draws on examples from ED triage. According to international literature, ED triage training time varies from less than an hour to 12 weeks (Butler et al. 2023). In the UK, NHS England requires appropriate training (NHS England 2022b, August 12), covering areas such as mild to acute care, patient assessment, pain management, medicines management, moving and handling, infection prevention and control, safeguarding children and adults, documentation and record‐keeping and managing violence and aggression (Royal College of Nursing 2017). To successfully implement a triage system in primary care, staff need to complete a minimum level of training beforehand, and a curriculum tailored to the primary care setting should be developed.

The use of triage patients' symptoms varied by age, time of day and urgency, suggesting triage must be flexible and sensitive to demographic and temporal factors. Notably, older and less affluent patients were less likely to use telephone triage services (Elliott et al. 2015), pointing to potential inequities in access. While generational shifts may eventually mitigate this gap, immediate strategies are needed to improve awareness and accessibility. To bridge the current gap, targeted promotion and education efforts could raise awareness and encourage usage among these populations. Furthermore, service developers and policymakers should explore strategies to improve access for these groups to ensure equitable healthcare delivery (Elliott et al. 2015).

Triage safety was a recurring concern. Evidence of mis‐triage or cautious over‐referrals raises important questions about accuracy and risk management (Elliott et al. 2015; Gallagher et al. 1998; Hodgins et al. 2022). While some studies demonstrated good alignment between nurse and GP triage decisions (Eminovic et al. 2004), high patients' satisfaction level (Gallagher et al. 1998), high sensitivity in identifying patients needing urgent care (Hodgins et al. 2022). To address concerns about mis‐triage and enhance accuracy, periodic monitoring, outcome evaluation and the development of decision support tools and clear guidelines are essential for ensuring patient safety and effective triage practices.

Studies on the economic impact of triage in primary care present mixed results. Some models, such as nurse‐led asthma triage, demonstrated cost savings (Gruffydd‐Jones et al. 2005), while others showed minimal or no difference in costs (Richards et al. 2002; Campbell et al. 2014). Comparisons between call center and clinic‐based triage offer additional insights. NHS Direct triage was longer, more costly and involved more referrals to GPs, as nurses handled fewer calls independently and spent significantly more time per patient. In contrast, clinic nurses—familiar with their patients—made quicker, more confident decisions, enhancing efficiency and reducing costs (Richards et al. 2004b). While some studies highlight potential economic benefits of triage, there is insufficient research to draw definitive conclusions. Further studies are needed to better understand the cost implications of triage in primary care.

### 4.1 Limitations

We used rigorous methods to identify the current evidence on triage systems in primary care. However, the generalizability of our findings is limited by the focus on studies conducted in the UK. This focus allowed for a deeper understanding of triage systems within the UK primary care context, where most community residents are registered under the National Health Service. We cannot speculate on the relevance of our results to settings where primary care approaches differ from those in the UK. Despite these limitations, we believe our findings will be of interest to many settings considering the introduction of primary care triage systems, particularly to address challenges such as GP shortages and unnecessary ED overcrowding. Future research could expand this work by exploring the use of triage systems in primary care across diverse international settings, without restricting the study region to a single country. Additionally, we employed narrative synthesis methods due to the variability among the included studies. While this approach allowed us to synthesise heterogeneous data, it relies on the reviewer's judgement in organising and interpreting findings, which can introduce potential bias. Moreover, we encountered challenges in merging and interpreting data derived from instruments with different measurement scales, which may have affected the consistency of our synthesis. Future research should aim to achieve homogeneity in study design, enabling sub‐analyses or meta‐analyses with reduced heterogeneity. Another limitation is that we did not include grey literature, which may introduce a potential bias. In future research, it is necessary to conduct a broader study that includes grey literature.

### 4.2 Implications for Practice and Research

Rising demand in primary care has led to challenges such as patient safety, staffing shortages, workload and costs. The implementation of triage systems in primary care can be a potential solution. However, gaps remain in understanding how triage systems are utilised in primary care, their benefits, challenges and cost‐effectiveness. This study is the first to comprehensively examine the overall use, benefits, challenges and effects of triage systems specifically within UK primary care. Our findings offer valuable insights into the application of triage systems, providing a foundation for better understanding their role in primary care.

To improve practice, there is a need for standardised decision support tools and clear clinical guidelines tailored to the primary care context. Training and education programmes should be implemented prior to role transitions to ensure that staff are adequately prepared for triage responsibilities. Decision support tools specifically developed for primary care could enhance the safety and consistency of triage, particularly in managing complex or uncertain cases. For future research, long‐term studies are needed to evaluate the implementation of triage in real‐world primary care settings and assess its impact on key outcomes, including health outcomes, workload redistribution, clinical effectiveness and patient safety over time. Additionally, robust economic evaluations are required to determine the cost‐effectiveness of various triage models. Exploring the experiences of vulnerable groups such as older adults or low‐income populations with triage services could also highlight potential equity issues and guide more inclusive service development.

## 5 Conclusion

Introducing a triage system in primary care offers new opportunities to enhance healthcare delivery. Through thorough review, we examined how primary care triage is currently implemented. Implementing triage in primary care can help address pressing challenges such as GP shortages, limited patient access to care and ED overcrowding. However, concerns still remain, including the risk of mis‐triage, the lack of standardised protocols or guidelines and insufficient training and education for triage personnel. Moreover, the cost‐effectiveness of triage remains uncertain. Developing effective triage services will require careful consideration of triage patterns, common patient symptoms and health outcomes highlighted in this study. Operational demands include specialised education and training, workflow adjustments, appropriate resource allocation, additional staffing, clear guidelines or protocols and a robust decision support tool, are essential for a successful triage system. This study provides key insights for healthcare practitioners, researchers and policymakers aiming to improve triage systems in primary care, ultimately contributing to more accessible and efficient healthcare delivery. These findings should serve as a catalyst for future research and policy changes aimed at optimising triage processes in primary care.

## Author Contributions

All authors participated in the study design, data acquisition, analysis, synthesis and interpretation processes, and all agreed on the final version of the study (Han Nah Park, Kyeongmin Lee and Carolyn Wallace).

## Conflicts of Interest

The authors declare no conflicts of interest.

## Data Availability

Research data are not available as this is a review article.

## Appendix 1 Search Strategy

### Search Date: 14/10/2024

**Table:**

Database (Platform)	Search	Results
Pubmed (National library of medicine)	(“Primary Health Care”[Majr:NoExp] OR “Access to Primary Care”[Mesh] OR “Primary Care Nursing”[Mesh] OR “Physicians, Primary Care”[Mesh] OR “Family Practice”[Mesh] OR “Physicians, Family”[Mesh] OR “primary care”[tiab] OR “family practice”[tiab] OR “family medicine”[tiab] OR “PCP”[tiab] OR “Primary care provider”[tiab] OR “family physician*”[tiab] OR “primary physician*”[tiab] OR “primary doctor*”[tiab] OR “general practi*”[tiab] OR “pharma*”[tiab] OR “nursing*”[tiab] OR “nurse practitioner*”[tiab] OR “physiotherap*”[tiab] OR “social work*”[tiab] OR “health visit*”[tiab]) **AND** (“Triage”[Mesh] OR triag*[tiab]) **AND** ((“United Kingdom”[Mesh] OR “United Kingdom”[tiab]) **OR** (“UK”[tiab] OR “Wales”[tiab] OR “England”[tiab] OR “Scotland”[tiab] OR “Northern Ireland”[tiab] “Great Britain”[tiab]))	339
CINAHL WITH FULLTEXT	1. MH (“Primary Health Care” OR “Access to Primary Care” OR “Primary Nursing” OR “Physicians, Primary Care” OR “Family Practice” OR “Physicians, Family”) OR TI (“primary care” OR “family practice” OR “family medicine” OR “PCP” OR “Primary care provider” OR “family physician*” OR “primary physician*” OR “primary doctor*” OR “general practi*” OR “pharma*” OR “nursing*” OR “nurse practitioner*” OR “physiotherap*” OR “social work*” OR “health visit*”) OR AB (“primary care” OR “family practice” OR “family medicine” OR “PCP” OR “Primary care provider” OR “family physician*” OR “primary physician*” OR “primary doctor*” OR “general practi*” OR “pharma*” OR “nursing*” OR “nurse practitioner*” OR “physiotherap*” OR “social work*” OR “health visit*”) 2. MH (“Triage”) **OR TI (**“triag*”) **OR AB** (“triag*”) 3. MH (“United Kingdom”) **OR TI** (“United Kingdom” OR “UK” OR “Wales” OR “England” OR “Scotland” OR “Northern Ireland” OR “Great Britain”) **OR AB** (“United Kingdom” OR “UK” OR “Wales” OR “England” OR “Scotland” OR “Northern Ireland” OR “Great Britain”) 4. S1 AND S2 AND S3	55
EMBASE	1. (‘primary health care’/exp. OR ‘primary care access’/exp. OR ‘primary nursing’/exp. OR ‘general practitioner’/exp. OR ‘family practice’/exp. OR ‘family physicians’/exp) OR (‘primary care’ OR ‘family practice’ OR ‘family medicine’ OR ‘PCP’ OR ‘Primary care provider’ OR ‘family physician*’ OR ‘primary physician*’ OR ‘primary doctor*’ OR ‘general practi*’ OR ‘pharmac*’ OR ‘nursing*’ OR ‘nurse practitioner*’ OR ‘physiotherap*’ OR ‘social work*’ OR ‘health visit*’):ti,ab,kw 2. ‘patient triage’/exp. OR (triag*):ti,ab,kw 3. ‘United Kingdom’/exp. OR (“United Kingdom*” OR UK OR Wales OR England OR Scotland OR “Northern Ireland” OR “Great Britain”):ti,ab,kw 4. #1 AND #2 AND #3	819
COCHRANE Library	1. [mh primary health care] OR (“primary care” OR ((family OR primary OR general) NEXT (practi* OR physician* OR medicine OR doctor*)) OR PCP OR pharmacy* OR nursing* OR (nurse NEXT practitioner*) OR physiotherapy* OR (social NEXT worker*) OR (health NEXT visiting*)):ti,ab,kw 2. [mh triage] OR (triag*):ti,ab,kw 3. [mh United Kingdom] OR (United NEXT Kingdom* OR UK OR Wales OR England OR Scotland OR Northern NEXT Ireland OR Great NEXT Britain):ti,ab,kw #1 AND #2 AND #3	227 (Cochrane reviews 126, Cochrane protocols 20, trials 75, special collections 1, clinical answers 5)
Total		1440

## References

[jan70037-bib-0001] Bunn, F. , G. Byrne , and S. Kendall . 2005. “The Effects of Telephone Consultation and Triage on Healthcare Use and Patient Satisfaction: A Systematic Review.” British Journal of General Practice: The Journal of the Royal College of General Practitioners 55, no. 521: 956–961. 16378566 PMC1570504

[jan70037-bib-0002] Butler, K. , N. Anderson , and A. Jull . 2023. “Evaluating the Effects of Triage Education on Triage Accuracy Within the Emergency Department: An Integrative Review.” International Emergency Nursing 70: 101322. 10.1016/j.ienj.2023.101322. 37597277

[jan70037-bib-0003] Calitri, R. , F. C. Warren , B. Wheeler , et al. 2015. “Distance From Practice Moderates the Relationship Between Patient Management Involving Nurse Telephone Triage Consulting and Patient Satisfaction With Care.” Health & Place 34: 92–96. 10.1016/j.healthplace.2015.04.002. 25982703

[jan70037-bib-0004] Campbell, J. L. , E. Fletcher , N. Britten , et al. 2014. “Telephone Triage for Management of Same‐Day Consultation Requests in General Practice (The ESTEEM Trial): A Cluster‐Randomised Controlled Trial and Cost‐Consequence Analysis.” Lancet (London, England) 384, no. 9957: 1859–1868. 10.1016/S0140-6736(14)61058-8. 25098487

[jan70037-bib-0005] Charles‐Jones, H. , C. May , J. Latimer , and M. Roland . 2003. “Telephone Triage by Nurses in Primary Care: What Is It for and What Are the Consequences Likely to Be?” Journal of Health Services Research & Policy 8, no. 3: 154–159. 10.1258/135581903322029502. 12869341

[jan70037-bib-0006] Covidence Systematic Review Software . 2025. “Veritas Health Innovation, Melbourne, Australia.” www.covidence.org.

[jan70037-bib-0007] Crouch, R. , H. Woodfield , J. Dale , and A. Patel . 1997. “Telephone Assessment and Advice: A Training Programme.” Nursing Standard (Royal College of Nursing (Great Britain): 1987) 11, no. 47: 41–44. 10.7748/ns.11.47.41.s46. 9355534

[jan70037-bib-0008] Dhollande, S. , A. Taylor , S. Meyer , and M. Scott . 2021. “Conducting Integrative Reviews: A Guide for Novice Nursing Researchers.” Journal of Research in Nursing 26, no. 5: 427–438. 10.1177/1744987121997907. 35251272 PMC8894639

[jan70037-bib-0009] Elliott, A. M. , A. McAteer , D. Heaney , L. D. Ritchie , and P. C. Hannaford . 2015. “Examining the Role of Scotland's Telephone Advice Service (NHS 24) for Managing Health in the Community: Analysis of Routinely Collected NHS 24 Data.” BMJ Open 5, no. 8: e007293. 10.1136/bmjopen-2014-007293. PMC4554912 26310396

[jan70037-bib-0010] Elliott, M. , S. Jones , C. Johnson , and C. Wallace . 2020. “What Are the Benefits of Nurse‐Led Triage in Primary Care?” Primary Health Care 30, no. 3: 28–34. 10.7748/phc.2020.e1607.

[jan70037-bib-0011] Eminovic, N. , J. C. Wyatt , A. M. Tarpey , G. Murray , and G. J. Ingrams . 2004. “First Evaluation of the NHS Direct Online Clinical Enquiry Service: A Nurse‐Led Web Chat Triage Service for the Public.” Journal of Medical Internet Research 6, no. 2: e17. 10.2196/jmir.6.2.e17. 15249266 PMC1550598

[jan70037-bib-0012] Erkelens, D. C. , F. H. Rutten , L. T. Wouters , et al. 2020. “Accuracy of Telephone Triage in Patients Suspected of Transient Ischaemic Attack or Stroke: A Cross‐Sectional Study.” BMC Family Practice 21, no. 1: 256. 10.1186/s12875-020-01334-3. 33278874 PMC7719259

[jan70037-bib-0013] Farrohknia, N. , M. Castrén , A. Ehrenberg , et al. 2011. “Emergency Department Triage Scales and Their Components: A Systematic Review of the Scientific Evidence.” Scandinavian Journal of Trauma, Resuscitation and Emergency Medicine 19: 42. 10.1186/1757-7241-19-42. 21718476 PMC3150303

[jan70037-bib-0014] Fekonja, Z. , S. Kmetec , U. Fekonja , N. Mlinar Reljić , M. Pajnkihar , and M. Strnad . 2023. “Factors Contributing to Patient Safety During Triage Process in the Emergency Department: A Systematic Review.” Journal of Clinical Nursing 32, no. 17–18: 5461–5477. 10.1111/jocn.16622. 36653922

[jan70037-bib-0015] Gallagher, M. , T. Huddart , and B. Henderson . 1998. “Telephone Triage of Acute Illness by a Practice Nurse in General Practice: Outcomes of Care.” British Journal of General Practice: The Journal of the Royal College of General Practitioners 48, no. 429: 1141–1145. 9667088 PMC1410031

[jan70037-bib-0016] Gruffydd‐Jones, K. , S. Hollinghurst , S. Ward , and G. Taylor . 2005. “Targeted Routine Asthma Care in General Practice Using Telephone Triage.” British Journal of General Practice: The Journal of the Royal College of General Practitioners 55, no. 521: 918–923. 16378560 PMC1570530

[jan70037-bib-0017] Hodgins, P. , M. McMinn , M. J. Reed , S. W. Mercer , and B. Guthrie . 2022. “Telephone Triage of Young Adults With Chest Pain: Population Analysis of NHS24 Calls in Scottish Unscheduled Care.” Emergency Medicine Journal: EMJ 39, no. 7: 508–514. 10.1136/emermed-2020-210594. 34675053

[jan70037-bib-0018] Holt, T. A. , E. Fletcher , F. Warren , et al. 2016. “Telephone Triage Systems in UK General Practice: Analysis of Consultation Duration During the Index Day in a Pragmatic Randomised Controlled Trial.” British Journal of General Practice: The Journal of the Royal College of General Practitioners 66, no. 644: e214–e218. 10.3399/bjgp16X684001. 26917660 PMC4758502

[jan70037-bib-0051] Hong, Q. N. , A. Gonzalez‐Reyes , and P. Pluye . 2018. “Improving the Usefulness of a Tool for Appraising the Quality of Qualitative, Quantitative and Mixed Methods Studies, the Mixed Methods Appraisal Tool (MMAT).” Journal of Evaluation in Clinical Practice 24, no. 3: 459–467. 10.1111/jep.12884. 29464873

[jan70037-bib-0019] Horton, M. , R. V. Harris , and R. S. Ireland . 2001. “The Development and Use of a Triage Protocol for Patients With Dental Problems Contacting an Out‐Of‐Hours General Medical Practitioner Cooperative.” Primary Dental Care: Journal of the Faculty of General Dental Practitioners (UK) 8, no. 3: 93–97. 10.1308/135576101322561886. 11490705

[jan70037-bib-0020] Jiwa, M. , N. Mathers , and M. Campbell . 2002. “The Effect of GP Telephone Triage on Numbers Seeking Same‐Day Appointments.” British Journal of General Practice: The Journal of the Royal College of General Practitioners 52, no. 478: 390–391. 12014537 PMC1314295

[jan70037-bib-0021] Jones, K. , P. Gilbert , J. Little , and K. Wilkinson . 1998. “Nurse Triage for House Call Requests in a Tyneside General Practice: Patients' Views and Effect on Doctor Workload.” British Journal of General Practice: The Journal of the Royal College of General Practitioners 48, no. 431: 1303–1306. 9747546 PMC1410152

[jan70037-bib-0022] Kontopantelis, E. , M. Roland , and D. Reeves . 2010. “Patient Experience of Access to Primary Care: Identification of Predictors in a National Patient Survey.” BMC Family Practice 11: 61. 10.1186/1471-2296-11-61. 20799981 PMC2936332

[jan70037-bib-0023] Lawson, E. 2022. “The Global Primary Care Crisis.” British Journal of General Practice 73, no. 726: 3. 10.3399/bjgp23X731469. PMC9799366 36543556

[jan70037-bib-0024] Lewis, J. , T. Stone , R. Simpson , et al. 2021. “Patient Compliance With NHS 111 Advice: Analysis of Adult Call and ED Attendance Data 2013‐2017.” PLoS One 16, no. 5: e0251362. 10.1371/journal.pone.0251362. 33970946 PMC8109810

[jan70037-bib-0025] Mayor, S. 2014. “Primary Care Telephone Triage Does Not Reduce Workload, Study Finds.” BMJ (Clinical Research Ed.) 349: g4958. 10.1136/bmj.g4958. 25092433

[jan70037-bib-0026] Michel, J. , A. Manns , S. Boudersa , et al. 2024. “Clinical Decision Support System in Emergency Telephone Triage: A Scoping Review of Technical Design, Implementation and Evaluation.” International Journal of Medical Informatics 184: 105347. 10.1016/j.ijmedinf.2024.105347. 38290244

[jan70037-bib-0027] Murdoch, J. , A. Varley , E. Fletcher , et al. 2015. “Implementing Telephone Triage in General Practice: A Process Evaluation of a Cluster Randomised Controlled Trial.” BMC Family Practice 16: 47. 10.1186/s12875-015-0263-4. 25887747 PMC4395901

[jan70037-bib-0028] Nakubulwa, M. A. , G. Greenfield , E. Pizzo , et al. 2022. “To What Extent Do Callers Follow the Advice Given by a Non‐Emergency Medical Helpline (NHS 111): A Retrospective Cohort Study.” PLoS One 17, no. 4: e0267052. 10.1371/journal.pone.0267052. 35446886 PMC9022858

[jan70037-bib-0030] NHS England . 2020. “Advice on How to Establish a Remote ‘Total Triage’ Model in General Practice Using Online Consultations.” https://www.england.nhs.uk/coronavirus/documents/advice‐on‐how‐to‐establish‐a‐remote‐total‐triage‐model‐in‐general‐practice‐using‐online‐consultations/.

[jan70037-bib-0032] NHS England . 2022a. “The Healthcare Ecosystem.” https://digital.nhs.uk/developer/guides‐and‐documentation/introduction‐to‐healthcare‐technology/the‐healthcare‐ecosystem.

[jan70037-bib-0031] NHS England . 2022b. “Guidance for Emergency Departments: Initial Assessment. NHS England.” https://www.england.nhs.uk/guidance‐for‐emergency‐departments‐initial‐assessment/.

[jan70037-bib-0034] NHS England . 2024a. “Primary Medical Services Policy and Guidance Manual (PGM) (Version 5).” https://www.england.nhs.uk/long‐read/primary‐medical‐services‐policy‐and‐guidance‐manual‐pgm/#part‐a‐excellent‐commissioning‐and‐partnership‐working.

[jan70037-bib-0035] NHS England . 2024b. “Digitally Enabled Triage.” https://digital.nhs.uk/developer/guides‐and‐documentation/introduction‐to‐healthcare‐technology/the‐healthcare‐ecosystem.

[jan70037-bib-0036] NHS England . 2024c. “NHS 111 Online.” https://digital.nhs.uk/services/nhs‐111‐online.

[jan70037-bib-0037] Orton, P. , C. Orton , and D. Pereira Gray . 2012. “Depersonalised Doctors: A Cross‐Sectional Study of 564 Doctors, 760 Consultations and 1876 Patient Reports in UK General Practice.” BMJ Open 2, no. 1: e000274. 10.1136/bmjopen-2011-000274. PMC3274717 22300669

[jan70037-bib-0038] Page, M. J. , J. E. McKenzie , P. M. Bossuyt , et al. 2021. “The PRISMA 2020 Statement: An Updated Guideline for Reporting Systematic Reviews.” BMJ (Clinical Research Ed.) 372: n71. 10.1136/bmj.n71. PMC8005924 33782057

[jan70037-bib-0039] Popay, J. , H. Roberts , A. Sowden , et al. 2006. “Guidance on the Conduct of Narrative Synthesis in Systematic Reviews.” A Product From the ESRC Methods Programme Version 1, no. 1: b92. https://citeseerx.ist.psu.edu/document?repid=rep1&type=pdf&doi=ed8b23836338f6fdea0cc55e161b0fc5805f9e27.

[jan70037-bib-0040] Purc‐Stephenson, R. J. , and C. Thrasher . 2012. “Patient Compliance With Telephone Triage Recommendations: A Meta‐Analytic Review.” Patient Education and Counseling 87, no. 2: 135–142. 10.1016/j.pec.2011.08.019. 22001679

[jan70037-bib-0043] Richards, D. A. , J. Meakins , J. Tawfik , et al. 2002. “Nurse Telephone Triage for Same Day Appointments in General Practice: Multiple Interrupted Time Series Trial of Effect on Workload and Costs.” BMJ (Clinical Research Ed.) 325, no. 7374: 1214. 10.1136/bmj.325.7374.1214. PMC135495 12446539

[jan70037-bib-0042] Richards, D. A. , J. Meakins , J. Tawfik , L. Godfrey , E. Dutton , and P. Heywood . 2004a. “Quality Monitoring of Nurse Telephone Triage: Pilot Study.” Journal of Advanced Nursing 47, no. 5: 551–560. 10.1111/j.1365-2648.2004.03132.x. 15312118

[jan70037-bib-0041] Richards, D. A. , L. Godfrey , J. Tawfik , et al. 2004b. “NHS Direct Versus General Practice Based Triage for Same Day Appointments in Primary Care: Cluster Randomised Controlled Trial.” BMJ (Clinical Research Ed.) 329, no. 7469: 774. 10.1136/bmj.38226.605995.55. PMC520999 15377574

[jan70037-bib-0044] Rolland, E. , K. M. Moore , V. A. Robinson , and D. McGuinness . 2006. “Using Ontario's “Telehealth” Health Telephone Helpline as an Early‐Warning System: A Study Protocol.” BMC Health Services Research 6: 10. 10.1186/1472-6963-6-10. 16480500 PMC1431529

[jan70037-bib-0045] Royal College of Nursing . 2017. National Curriculum and Competency Framework: Emergency Nursing (Level 1). Royal College of Nursing. https://www.rcn.org.uk/professional‐development/publications/PUB‐005883.

[jan70037-bib-0046] St George, I. , M. Cullen , L. Gardiner , and G. Karabatsos . 2008. “Universal Telenursing Triage in Australia and New Zealand ‐ a New Primary Health Service.” Australian Family Physician 37, no. 6: 476–479. 18523705

[jan70037-bib-0048] Warren, F. C. , R. Calitri , E. Fletcher , et al. 2015. “Exploring Demographic and Lifestyle Associations With Patient Experience Following Telephone Triage by a Primary Care Doctor or Nurse: Secondary Analyses From a Cluster Randomised Controlled Trial.” BMJ Quality and Safety 24, no. 9: 572–582. 10.1136/bmjqs-2015-003937. PMC4552919 25986572

[jan70037-bib-0049] Whittemore, R. , and K. Knafl . 2005. “The Integrative Review: Updated Methodology.” Journal of Advanced Nursing 52, no. 5: 546–553. 16268861 10.1111/j.1365-2648.2005.03621.x

[jan70037-bib-0050] World Health Organization . 2024. “Primary Care.” https://www.who.int/teams/integrated‐health‐services/clinical‐services‐and‐systems/primary‐care.

